# Comprehensive utilization of waste hemicelluloses during ethanol production to increase lactic acid yield: from pretreatment to fermentation

**DOI:** 10.1186/s13068-014-0189-4

**Published:** 2014-12-31

**Authors:** Liming Zhang, Tingting You, Lu Zhang, Mingfei Li, Feng Xu

**Affiliations:** Beijing Key Laboratory of Lignocellulosic Chemistry, Beijing Forestry University, Beijing, 100083 China; MOE Key Laboratory of Wooden Material Science and Application, Beijing Forestry University, Beijing, 100083 China

**Keywords:** Alkaline peroxide pretreatment, Hemicelluloses, Ethanol, Lactic acid, Cost-effective

## Abstract

**Background:**

Reducing the cost of producing cellulosic ethanol is essential for the industrialization of biorefinery. Several processes are currently under investigation, but few of these techniques are entirely satisfactory in terms of competitive cost or environmental impact. In this study, a new ethanol and lactic acid (LA) coproduction is proposed. The technique involved addition of waste alkaline peroxide pretreated hydrolysate (mainly LA and hemicelluloses) to the reaction mixture after ethanol fermentation (mainly LA and xylose) to reduce the ethanol production cost.

**Results:**

The following processes were investigated to optimize LA production: no addition of hemicelluloses or hydrolysate, addition of recycled hemicelluloses, and addition of concentrated hydrolysate. The addition of concentrated hydrolysate at 48 hours, which resulted in a maximum LA concentration of 22.3 g/L, was the most environment-friendly and cost-effective process. After the improved fermentation, 361 mg LA and 132 mg ethanol were produced from 1 g of raw poplar wood. That is, the production of one gallon of ethanol produced $9 worth of LA.

**Conclusions:**

The amount of LA produced from the pretreated hydrolysate and reaction mixture after ethanol fermentation cannot be underestimated. The recovery of hydrolysate rich in LA and hemicelluloses (or xylose) significantly improved LA yield and further reduced the ethanol production cost.

**Electronic supplementary material:**

The online version of this article (doi:10.1186/s13068-014-0189-4) contains supplementary material, which is available to authorized users.

## Background

Bioethanol as an alternative renewable energy source is receiving increasing attention worldwide because of the rapid accumulation of atmospheric greenhouse gases and the imminent depletion of fossil fuel reserves [1]. However, reducing cost for producing cellulosic ethanol remains the largest obstacle to emerging biorefinery [2]. The clear goal of making this process cost-competitive in today’s markets drives the continued pursuit of comprehensive utilization of cellulose, hemicelluloses, and lignin from biomass.

Hemicelluloses are being discarded in most cellulosic ethanol pilot and demonstration plants worldwide [3,4]. A previous study on hemicellulose conversion to ethanol has revealed that traditional *Saccharomyces cerevisiae* and *Zymomonas mobilis* fail to ferment the monosaccharides of hemicelluloses (such as xylose, arabinose, and rhamnose) to ethanol [5]. Some bacteria, such as *Escherichia coli*, *Klebsiella*, *Envinia*, *Bacillus*, and *Clostridia*, can utilize mixed hemicellulose sugars but either cannot produce ethanol or can only produce a limited quantity of ethanol [3]. Although hemicelluloses are difficult to ferment into ethanol, they are easily converted into lactic acid (LA) by biological fermentation [6-8] or catalytic conversion [9]. LA and poly-LA are valuable green chemicals with several industrial applications [10,11]. Additionally, the price of LA is almost twice that of ethanol [12]. Hence, the conversion of waste hemicellulose sugars to LA is essential to reduce the bioethanol cost.

Hemicelluloses are prone to degradation to LA during pretreatment, which is necessary for cellulosic ethanol bioconversion. Alvarez-Vasco and Zhang [13] found that LA was the most predominant product from alkaline peroxide (AP) pretreatment at temperatures above 140°C (approximately 6%). LA is also the main by-product of ethanol fermentation, and its concentration reached up to 10 g/L in simultaneous saccharification and fermentation in the authors’ previous study [14]. If the LA generated during pretreatment and ethanol fermentation is recovered, the biomass ethanol cost could be reduced significantly.

This work was designed to develop an innovative and economical LA and ethanol coproduction process from poplar by recovering the AP-pretreated hydrolysate and reaction mixture after ethanol fermentation, which are rich in hemicelluloses and LA. *Sacrau* poplar was pretreated with alkaline hydrogen peroxide at temperatures of 120 to 180°C with light H_2_O_2_ concentrations of 10% (g_H2O2_/g_wood_) in 20% NaOH (g_NaOH_/g_wood_). The hemicelluloses or concentrated hydrolysate were recovered and added to the reaction mixture after ethanol production to produce LA. Different parameters that affect LA yield, including pretreatment temperature, fermentation time of LA bacteria, and addition of hemicelluloses or concentrated hydrolysate, were examined.

## Results and discussion

### Component analysis of pretreated hydrolysate

AP pretreatment efficiently delignifies lignocellulose by disrupting the ester bonds between xylan and lignin, leading to the dissolution of hemicelluloses and lignin into pretreated hydrolysate [15]. To determine the amount of hemicelluloses in the pretreated hydrolysate, hemicellulose fractions were recovered and the monosaccharide compositions were analyzed as shown in Additional file 1: Table S1. The pretreatment of poplar with alkaline peroxide at 120, 140, 160, and 180°C for two hours removed 17.3%, 27.4%, 41.8%, and 41.9% (% of the initial amount of hemicelluloses in untreated poplar, w/w), respectively. However, the recovery rate was approximately 80% of the removed hemicelluloses. In all recycled hemicelluloses samples, xylose was the major sugar (82.3 to 86.7%), followed by glucose (5.3 to 5.5%), and galactose (1.4 to 1.7%). The precipitated hemicelluloses from the pretreated hydrolysate after concentration showed black color (Additional file 1: Figure S1). To observe the structural changes of hemicelluloses after pretreatment, the Fourier Transform Infrared (FI-IR) spectra of the recycled hemicelluloses (H_4_, 180°C) and typical alkaline-extracted hemicelluloses (H_0_) were recorded as shown in Additional file 1: Figure S2. The intensity of the band at 1,598 cm^−1^ varied among samples and increased from the brighter sample (H_0_) to the darker sample treated at 180°C [16]. This band was assigned to C = O groups in the lignin content, which presents a close correlation with the color of the material. Besides the lignin, the dark color of the material was probably due to pseudo-lignin or hexenuronic acids and chromophores produced during the oxidative processes [17]. Despite this difference, the recycled hemicelluloses sample showed a typical FT-IR spectra (1,200 to 800 cm^−1^) of hemicelluloses, without any significant differences after AP treatments [18,19].

**Figure 1:**
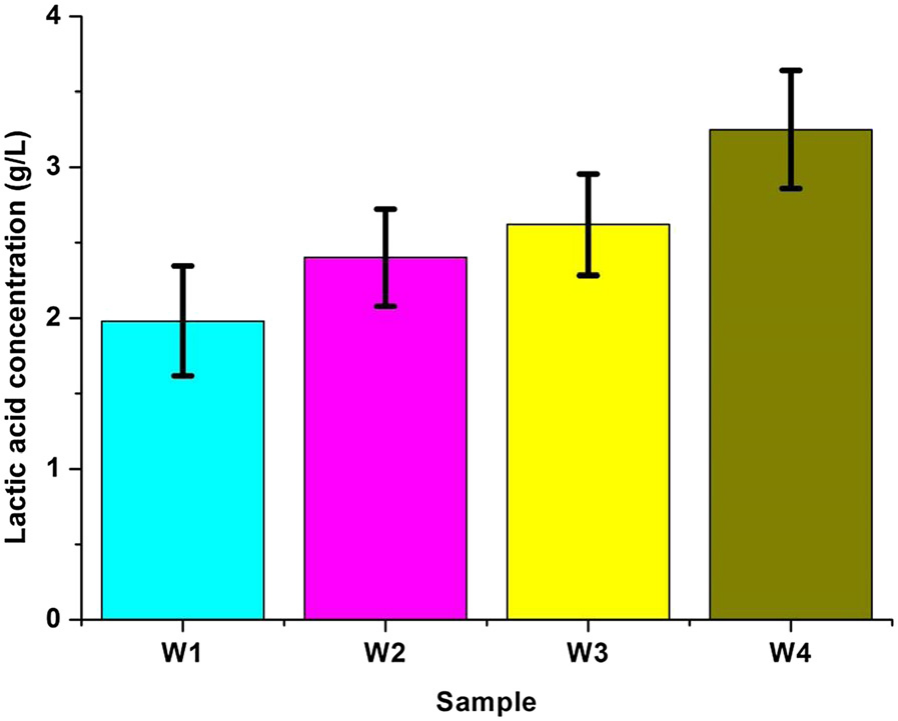
**Lactic acid concentration of samples treated at different temperatures.** W_1_, W_2_, W_3_, and W_4_ were samples treated at 120°C, 140°C, 160°C, and 180°C, respectively.

As shown in Additional file 1: Table S1, the hemicelluloses recovery was only approximately 80%, with up to 20% of the original xylan lost to degradation products through an endwise depolymerization or peeling reaction under alkaline condition [13]. The LA concentration in the pretreated hydrolysate was positively correlated with temperature (Figure 1). A maximum LA concentration of 3.3 g/L was observed when the poplar was pretreated at 180°C. This concentration is lower than that reported in a recent investigation [13], in which LA was the predominant product (approximately 6 g/L) from AP pretreated softwood above 140°C. The low LA concentration in the current study may be attributed to the lower susceptibility to alkali of the hardwood hemicelluloses (xylan) than the glucomannan of softwood [13]. The hemicellulose degradation in hardwood was not as severe as that in softwood, but the LA produced from the pretreatment hydrolysate cannot be underestimated from an economic standpoint.

### Effect of alkaline peroxide pretreatment conditions on lactic acid concentration

**Figure 2:**
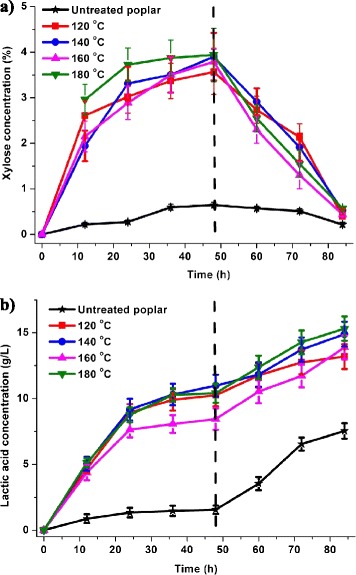
**Xylose (a) and lactic acid (b) concentration changes during 84-hour lactic acid fermentation.**

During the 48-hour ethanol fermentation, cellulose was degraded to monosaccharides, which were further converted to ethanol, LA, and other by-products by *S. cerevisiae* [20]. The xylan that remained in the pretreated poplar was also degraded by the commercial hemicellulases during the 48-hour ethanol fermentation. Since *S. cerevisiae* cells are unable to ferment xylose to ethanol, the xylose concentration continued to increase moderately. The highest xylose concentration reached 3.9 g/L (Figure 2a), which indicated that approximately 50% of the residual xylan in the pretreated samples was hydrolyzed into xylose after 48 hours of fermentation. Adding LA bacteria caused gradual xylose degradation, which resulted in decreased xylose concentration in the hydrolysate. Xylose concentration was less than 1 g/L at the end of fermentation.

With xylose consumption, the LA concentration increased significantly (*P* <0.05). The untreated sample achieved 7.5 g/L of LA after 84 hours of fermentation, whereas the LA concentration of W_4_ (180°C) was greatly improved to 15.3 g/L (Figure 2b). The improvement in the LA concentration of the AP-pretreated poplar compared with that of the untreated feedstock was largely attributed to the increase in polysaccharides accessibility because of the partial removal of hemicelluloses and lignin. However, the LA concentration of the samples pretreated at a high temperature was only slightly higher than that of the samples treated at a moderate temperature (from 13.2 to 15.3 g/L). The similarity in the LA concentration between the four treated samples could be attributed to their similar compositions because most of the cellulose was consumed by cellulase and *S. cerevisiae* in the first 48 hours of ethanol fermentation. The LA concentration of the pretreated biomass improved slightly with increasing pretreatment severity in this study, but the practical pretreatment conditions should be chosen comprehensively. Various factors should be considered aside from LA concentration, such as ethanol yield, pretreatment cost, and energy consumption. The sample synthesized under these conditions at 160°C was advantageous because of its high ethanol concentration (8.5 g/L; Additional file 1: Figure S3a) and low pretreatment temperature.

### Addition of recycled hemicelluloses and concentrated hydrolysate

**Figure 3:**
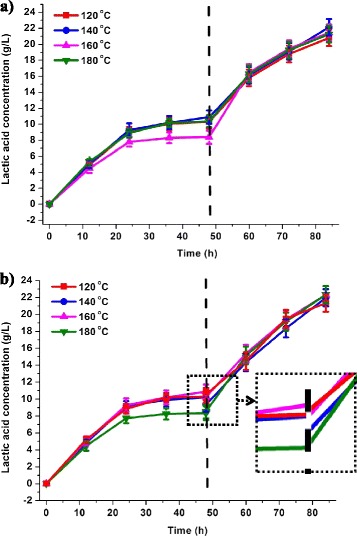
**Comparison of the addition of recycled hemicelluloses (a) and concentrated hydrolysate (b).**

The addition of recycled hemicelluloses remarkably increased LA concentration. Figure 3a shows that the LA concentration of W_3_ (160°C) was 8.4 g/L after 48 hours of ethanol fermentation. The LA concentration increased to 21.4 g/L (160°C) when the recycled hemicelluloses and LA bacteria were added to the solution. The LA was produced initially by the consumption of glucose in the reaction mixture [21]. With glucose consumption, the fermentable sugar was provided by hemicelluloses. Consequently, the final LA concentration of the samples depended largely on the amount of xylan that survived in the reaction mixture. Thus, the sample to which recycled hemicelluloses were added acquired higher LA concentration (20.8 to 22.1 g/L) than the sample without hemicelluloses (7.5 to 15.3 g/L, Figure 2b).

As shown in Figure 3b, LA production was lower in the sample with concentrated hydrolysate than that with recovered hemicelluloses during the initial stage. This phenomenon was due to the fact that the addition of 5 mL of concentrated hydrolysate resulted in the dilution of the LA concentration. However, in the subsequent fermentation, the low LA concentration in the reaction mixture slightly inhibited LA fermentation. Thus, the LA conversion of the concentrated hydrolysate achieved the best results of 22.3 g/L (sample treated at 160°C) at the end of fermentation. The addition of hemicelluloses and concentrated hydrolysate had equivalent LA concentrations (21.4 g/L and 22.3 g/L, respectively), but the LA productivity of concentrated hydrolysate was much higher than that of hemicelluloses because of the larger volume of fermentation liquor. Additionally, the precipitation of the hemicelluloses by acids and alcohols to obtain a certain degree of purification remains expensive because it requires several volumes of alcohol. Therefore, the concentrated hydrolysate addition was appropriate in obtaining the maximum LA yields from fermentation.

### Optimization of the addition time of lactic acid bacteria and concentrated hydrolysate

**Figure 4:**
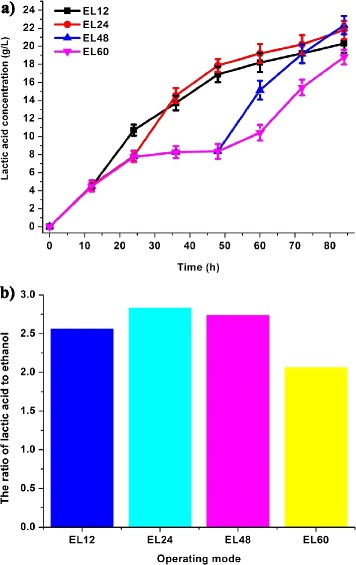
**Lactic acid concentration (a) and lactic acid-to-ethanol concentration ratio (b) changes when adding concentrated hydrolysate at different times.**

The following four cases were examined: 12 hours ethanol fermentation followed by 72 hours LA fermentation (EL12), 24 hours ethanol fermentation followed by 60 hours LA fermentation (EL24), 48 hours ethanol fermentation followed by 36 hours LA fermentation (EL48), and 60 hours ethanol fermentation followed by 24 hours LA fermentation (EL60). The optimal LA productivities for the EL12, EL24, EL48, and EL60 were 20.3 g/L, 21.8 g/L, 22.3 g/L, and 18.8 g/L, respectively (Figure 4a). The LA concentrations rapidly increased from EL12 to EL24, from EL24 to EL48, and then leveled off. The productivity of EL12 was lower than that of EL24 because the LA generated during ethanol fermentation was reduced. The equivalent LA productivities of EL24 and EL48 were due to the appropriate LA and ethanol fermentation time. When a typical fermentation time of 48 hours was increased to 60 hours, the LA concentration decreased from 22.3 to 18.8 g/L. This phenomenon could be explained as follows: 1) the inhibitors that adversely affect LA fermentation accumulated with the extension of ethanol fermentation time [20], 2) the time was too short for LA bacteria to produce LA, and 3) the remaining glucose concentration of EL60 was lower than that of EL48.

The final LA-to-ethanol ratios of EL12, EL24, EL48, and EL60 are shown in Figure 4b. The highest LA-to-ethanol ratio was EL24 (2:8), followed by EL48 (2:6). The LA-to-ethanol ratios of EL24 to EL48 were very similar, but the corresponding ethanol concentration of EL48 (8.5 g/L, 55.1%) was higher than those of other operation modes (Additional file 1: Figure S3b). Thus, EL48 was the most appropriate operation mode from the high-efficiency bioethanol conversion perspective.

### Calculation and mass balance

**Figure 5:**
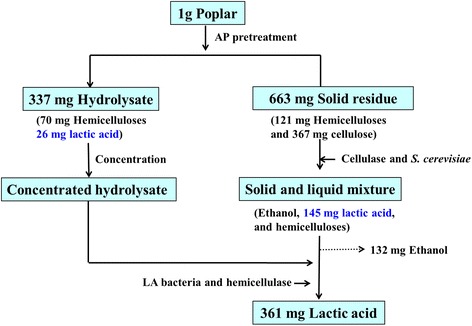
**Mass balance based on 1 g of dry poplar.** AP, Alkaline peroxide; LA, Lactic acid.

To provide an overview of the cellulose, hemicelluloses, and LA conversion from pretreatment to fermentation, an overall mass balance diagram based on 1 g of dry poplar with addition of concentrated hydrolysate at 48 hours is shown in Figure 5. After AP pretreatment, 121 mg or 208 mg of hemicelluloses were kept in the solid residue or dissolved in the pretreated hydrolysate, respectively. Additionally, 20% of the dissolved hemicelluloses in the hydrolysate were converted to LA (26 mg). The solid residue was used for ethanol fermentation, and 132 mg of ethanol and 145 mg of LA were acquired simultaneously after 48 hours of fermentation. The reaction mixture after ethanol fermentation was then subjected to LA production with the following preparation for up to 36 hours: 5 mL of concentrated hydrolysate, 50 IU/g of hemicellulase, and 0.05 g/g of LA bacteria. The hemicelluloses in the concentrated hydrolysate were converted into xylose by enzymes and then degraded by LA bacteria to produce 216 mg LA. After the improved fermentation, a total of 132 mg ethanol and 361 mg LA were produced. This integrated process increased the LA yield significantly because of the three-step LA enrichment process, namely, AP pretreatment, 48 hours of ethanol fermentation, and 36 hours of LA fermentation. Assuming an ethanol price of $1.65 per gallon (US), and LA cost of 50¢ per pound (US) [12], the production of 1 gallon of ethanol will produce $9 (US) worth of LA. The subsidy for cellulosic ethanol provided by the Farm Bill of 2008 was $1.01 (US) per gallon [22], which is lower than the additional value of LA. The hemicelluloses, lignin, and final fibrous residue after ethanol and LA coproduction can be converted to various materials, such as membrane material [23], polyurethane [24], and electricity [25]. If this comprehensive utilization is promoted, bioethanol production can be industrialized without government subsidies.

## Conclusions

This study clearly shows the importance of the comprehensive utilization of waste hemicelluloses during ethanol production to increase LA yield. LA production was significantly increased to 22.3 g/L by the three-step LA enrichment process, namely, AP pretreatment, 48 hours of ethanol fermentation, and 36 hours of LA production. The additional value of LA is $9 (US) per gallon of ethanol production, which is highly favorable from an economic point of view. Promoting the coproduction of LA and ethanol is a good way to implement the industrial production of biomass ethanol.

## Methods

### Lignocellulosic material, enzymes, and microorganism

*Sacrau* poplar was obtained from the experimental farm of Beijing Forestry University, China. The cellulase and hemicellulase used in this work were kindly supplied by Shanghai Youtell Biochemical Ltd. (Shanghai, China) and the filter paper activity (FPU) was 145 FPU/g and 100,000 IU/g, respectively. The microorganisms used for LA fermentation were freeze-dried LA bacteria (Beijing Chuanxiu Science and Technology Ltd., Beijing, China) that mainly comprised of *Lactobacillus plantarum*, *Lactobacillus bulgaricus*, and *Streptococcus thermophilus*. The bacteria were precultured at 40°C for 60 minutes with 2% glucose before fermentation. All other reagents were bought from Beijing Chemical Plant (Beijing, China).

### Alkaline peroxide pretreatment

**Figure 6:**
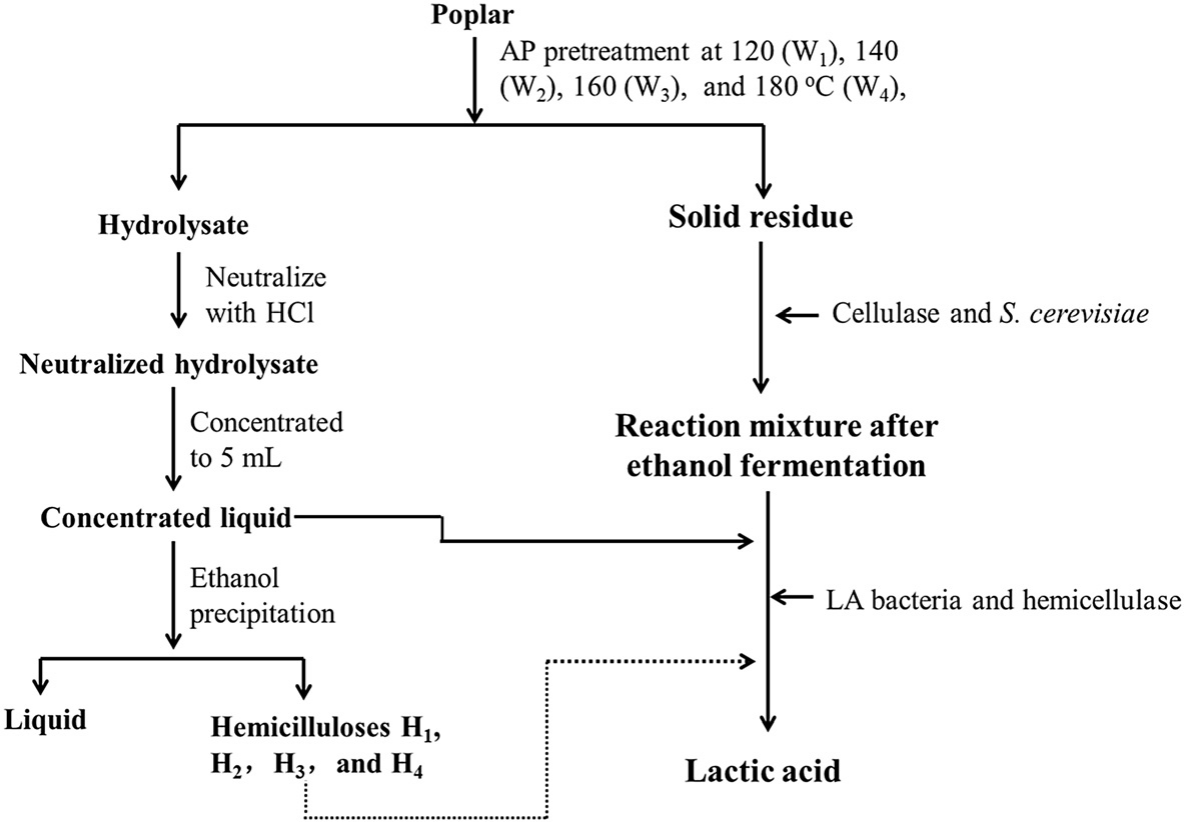
**Integrated ethanol and lactic acid coproduction process.**

The scheme for pretreatment, ethanol production, and LA fermentation of poplar is illustrated in Figure 6. AP pretreatments were performed at 120°C, 140°C, 160°C, and 180°C (in an oil bath, Xinyijia Chemical Co., Ltd., Jinan, China) with peroxide concentrations of 10% (g_H2O2_/g_wood_) and NaOH concentrations of 20% (g_NaOH_/g_wood_), respectively. After pretreatment, the solid phase (cellulosic residues) and liquid phase (pretreated hydrolysate) were separated by filtration under vacuum with a 400-mesh filter cloth (Beijing Chemical Plant, Beijing, China). The cellulosic residues were dried in an oven at 60°C until a constant weight and labeled as W_1_ (120°C), W_2_ (140°C), W_3_ (160°C), and W_4_ (180°C). The hydrolysate was neutralized with hydrochloric acid to a pH of 7 and concentrated to a volume of 5 mL. Lignin and hemicelluloses in the hydrolysate were precipitated by a previously published method [23]. LA concentration of the hydrolysate was determined by a high-performance liquid chromatography (HPLC) system (Agilent 1200 series, Agilent Technologies, California, United States) using an AMINEX HPX-87H column (8 mm, Bio-Rad Laboratories, California, United States) with a refractive index detector (Agilent Technologies, California, United States). The column temperature was 30°C and the flow rate of the eluent (5 mmol H_2_SO_4_) was 0.6 mL min^-1^. All measurements were performed in triplicate.

### Ethanol production and lactic acid fermentation

For ethanol fermentation, 1 g of cellulosic residue was added to 20 mL (5%, w/v) of 50 mM sodium acetate buffer [14]. After ethanol fermentation, the reaction mixture was sterilized with ultraviolet lamp (Ziguang, Electric Co., Ltd., Nanjing, China) to remove the effect of *S. cerevisiae*. LA fermentation was conducted by combining 0.05 g dry LA bacteria cells and 50 IU hemicellulase per gram of substrate at 48 hours fermentation, and then the mixture was incubated in a shaker at 43°C and 100 rpm for 36 hours. The following processes were investigated to optimize LA production: no addition of hemicelluloses or hydrolysate, addition of recycled hemicelluloses, and addition of concentrated hydrolysate. The effect of LA bacteria addition time on the LA yield and the LA-to-ethanol ratio was also discussed.

## Additional file

Additional file 1:
**Supplementary supporting data.** **Table S1.** Hemicelluloses removal, recovered hemicelluloses yield, and sugar component analysis of recovered hemicelluloses. **Figure S1.** The hemicelluloses recovered from pretreated hydrolysate at 180°C (H4) and typical alkaline hemicelluloses (H_0_). **Figure S2.** The FT-IR spectra of recovered hemicelluloses (H_4_) and typical alkaline hemicelluloses (H_0_). **Figure S3.** (a) Ethanol concentration of samples treated at different temperature after 48-h ethanol fermentation; (b) final ethanol concentration of EL12, EL24, EL48, and EL60.

## Abbreviations

AP: alkaline peroxide
EL12: 12 hours of ethanol fermentation followed by 70 hours of LA fermentation
EL24: 24 hours of ethanol fermentation followed by 60 hours of LA fermentation
EL48: 48 hours of ethanol fermentation followed by 36 hours of LA fermentation
EL60: 60 hours of ethanol fermentation followed by 24 hours of LA fermentation
LA: lactic acid
FI-IR: Fourier Transform Infrared
HPLC: High-performance Liquid Chromatography
