# Supplementary material for: Comprehensive utilization of waste hemicelluloses during ethanol production to increase lactic acid yield: from pretreatment to fermentation
Source: Biotechnol Biofuels. 2014 Dec 31;7:494. doi: 10.1186/s13068-014-0189-4 (PMC4300168; doi:10.1186/s13068-014-0189-4)
Supplement: Additional file 1: — Supplementary supporting data. Table S1. Hemicelluloses removal, recovered hemicelluloses yield, and sugar component analysis of recovered hemicelluloses. Figure S1. The hemicelluloses recovered from pretreated hydrolysate at 180°C (H4) and typical alkaline hemicelluloses (H0). Figure S2. The FT-IR spectra of recovered hemicelluloses (H4) and typical alkaline hemicelluloses (H0). Figure S3. (a) Ethanol concentration of samples treated at different temperature after 48-h ethanol fermentation; (b) final ethanol concentration of EL12, EL24, EL48, and EL60. [file 13068_2014_189_MOESM1_ESM.docx]

**Comprehensive utilization of waste hemicelluloses during ethanol production to increase lactic acid yield: from pretreatment to fermentation**

Liming Zhang^1^, Tingting You^1^, Lu Zhang^1^, Mingfei Li ^1,2^, Feng Xu^1,2§^

^1^ Beijing Key Laboratory of Lignocellulosic Chemistry, Beijing Forestry University, Beijing, 100083, China

^2^ MOE Key Laboratory of Wooden Material Science and Application, Beijing Forestry University, Beijing, 100083, China

^§^Corresponding author

Email address:

FX: [xfx315@bjfu.edu.cn](mailto:xfx315@bjfu.edu.cn)

# Additional files

### Supplementary Table S1 –Hemicelluloses removal, recovered hemicelluloses yield, and sugar component analysis of recovered hemicelluloses.

| **No.** | **Hemicelluloses removal (%) ^a^** | **Recovered hemicelluloses (%) ^a^** | **Rha (%) ^b^** | **Ara (%) ^b^** | **Gal (%) ^b^** | **Glu (%) ^b^** | **Xyl (%) ^b^** |
| --- | --- | --- | --- | --- | --- | --- | --- |
| **H1** | 17.3 | 14.2 | 1.8 | 1.4 | 1.7 | 5.5 | 82.3 |
| **H2** | 27.4 | 22.3 | 1.7 | 1.0 | 1.6 | 5.4 | 86.3 |
| **H3** | 41.8 | 33.4 | 1.7 | 1.1 | 1.4 | 5.3 | 86.5 |
| **H4** | 41.9 | 32.7 | 1.6 | 1.2 | 1.6 | 5.5 | 86.7 |

### a Wt.% of the initial amount of hemicelluloses in untreated poplar.

### b Wt.% of the recovered hemicelluloses.

### Supplementary Figure S1 –The hemicelluloses recovered from pretreated liquid at 180 ^o^C (H_4_) and typical alkaline hemicelluloses (H_0_).

**H_0_**


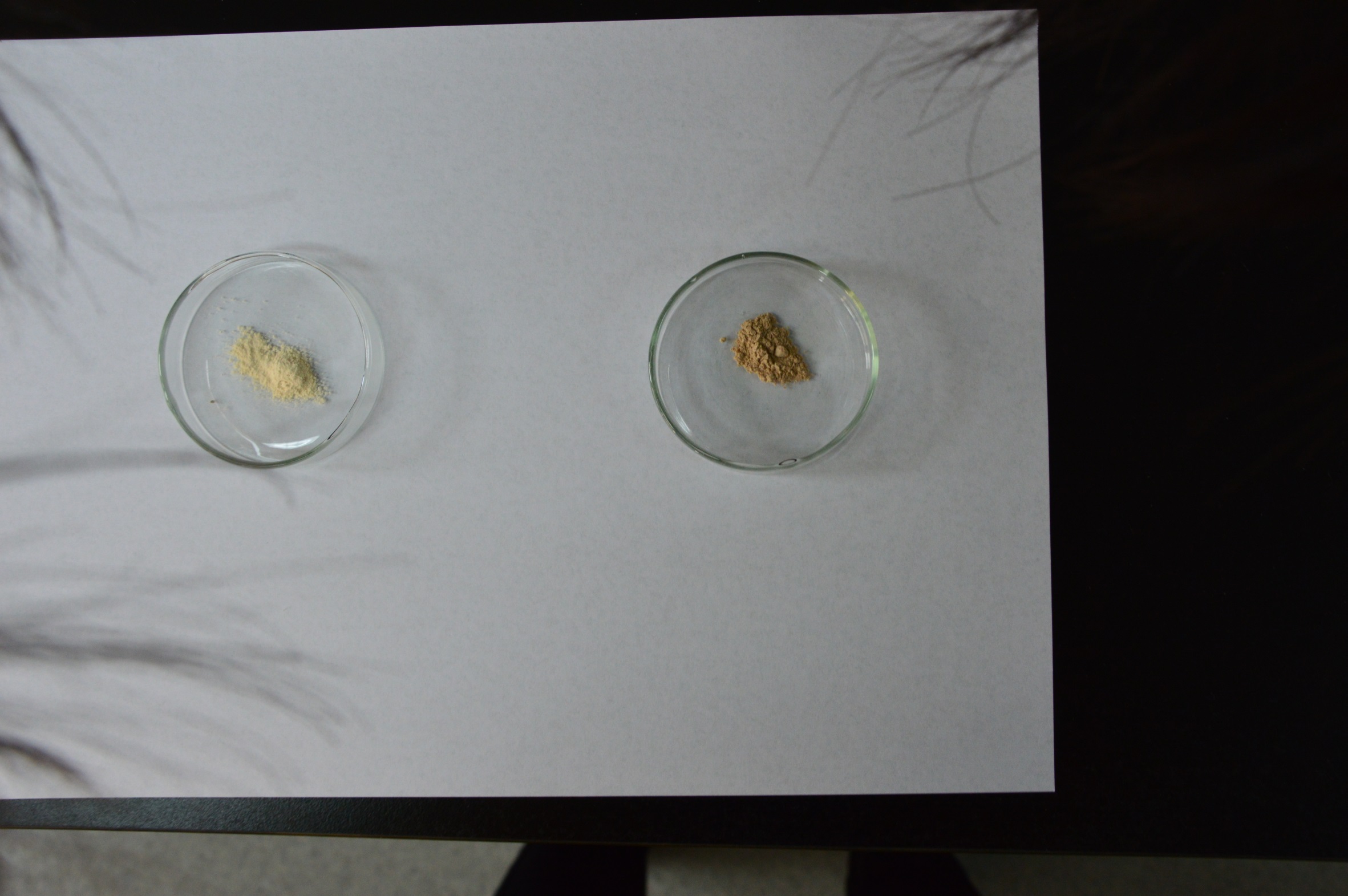

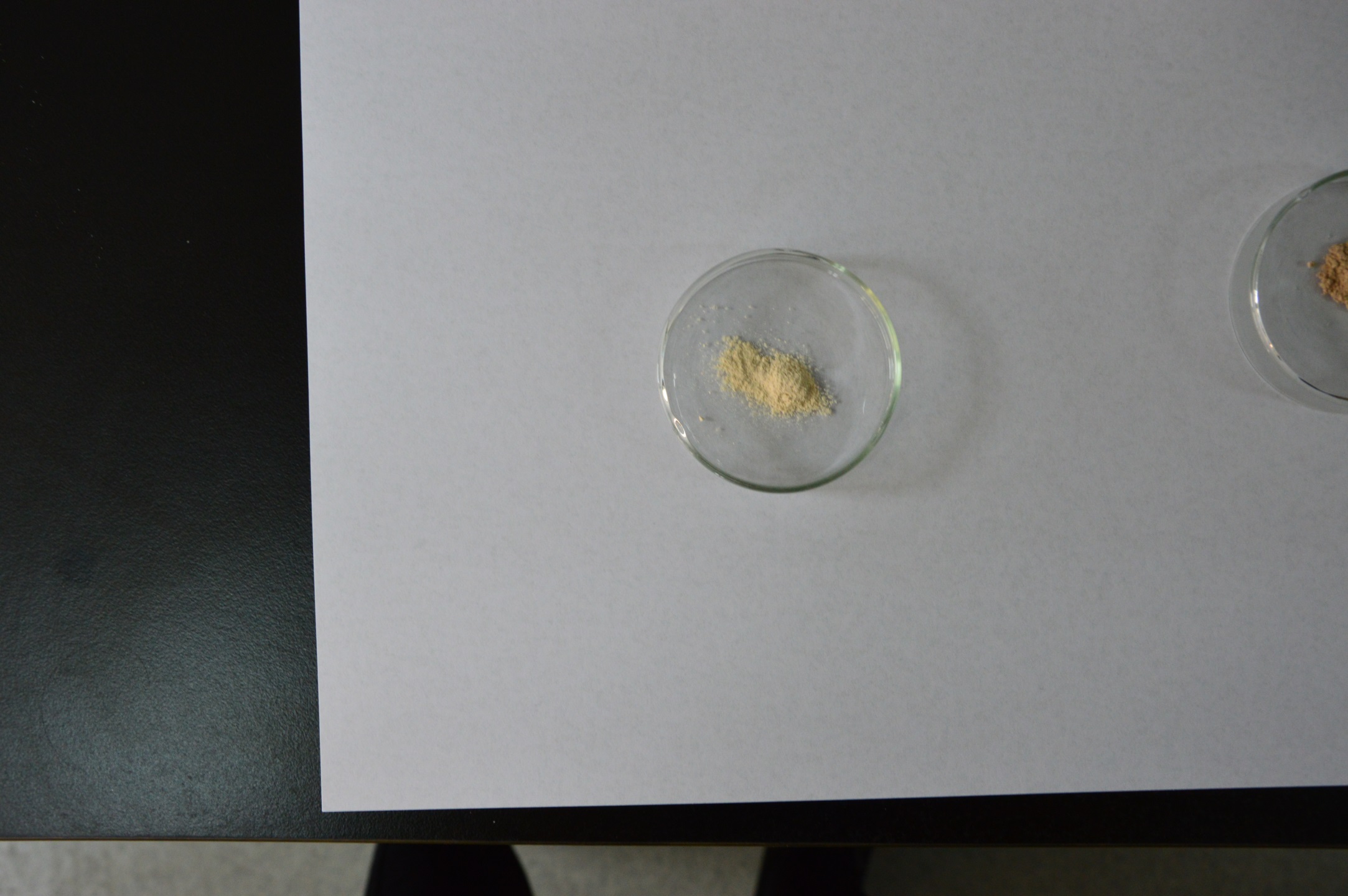


**H_4_**

### Supplementary Figure S2 –The FT-IR spectra of recovered hemicelluloses (H_4_) and typical alkaline hemicelluloses (H_0_).


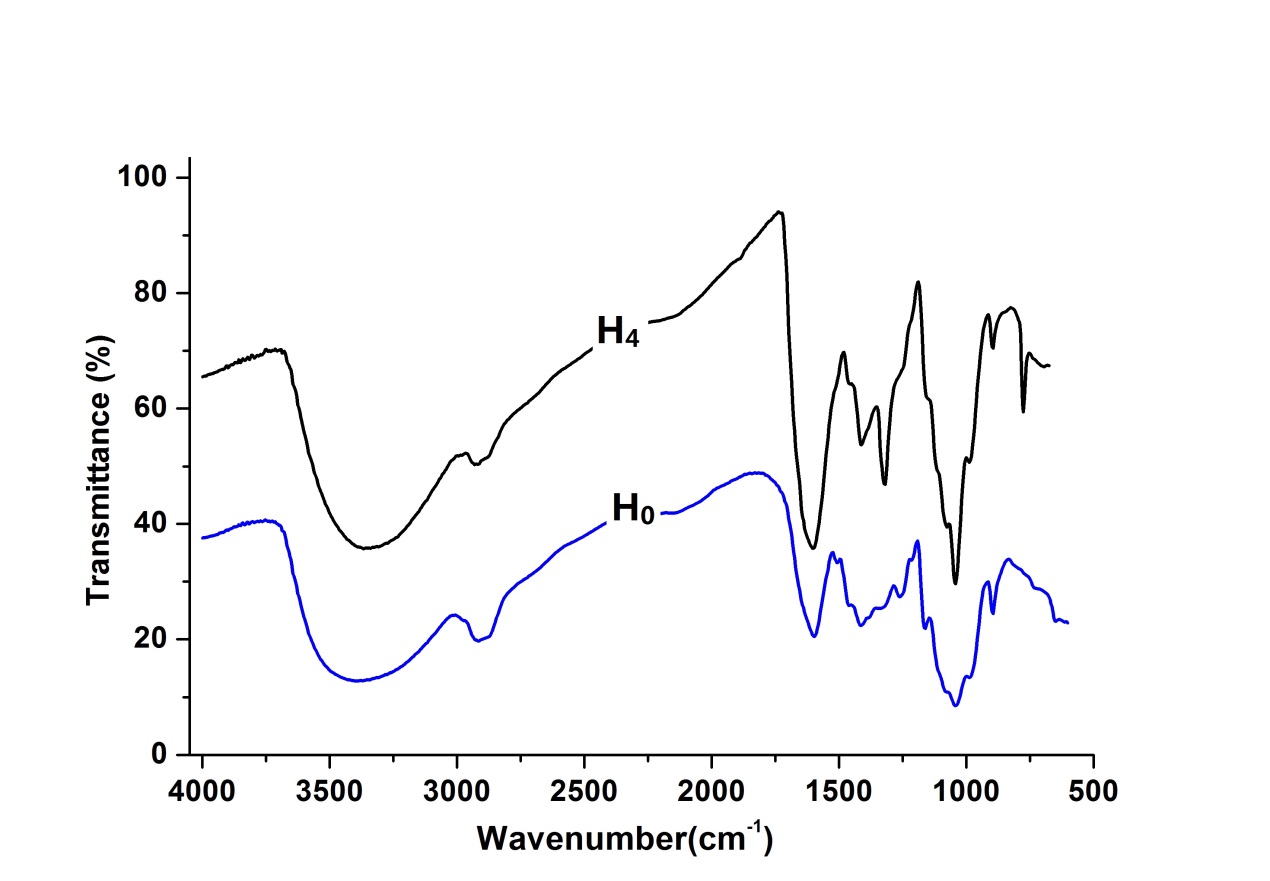


### Supplementary Figure S3 – (a) Ethanol concentration of samples treated at different temperature after 48-h ethanol fermentation; (b) final ethanol concentration of EL12, EL24, EL48, and EL60.


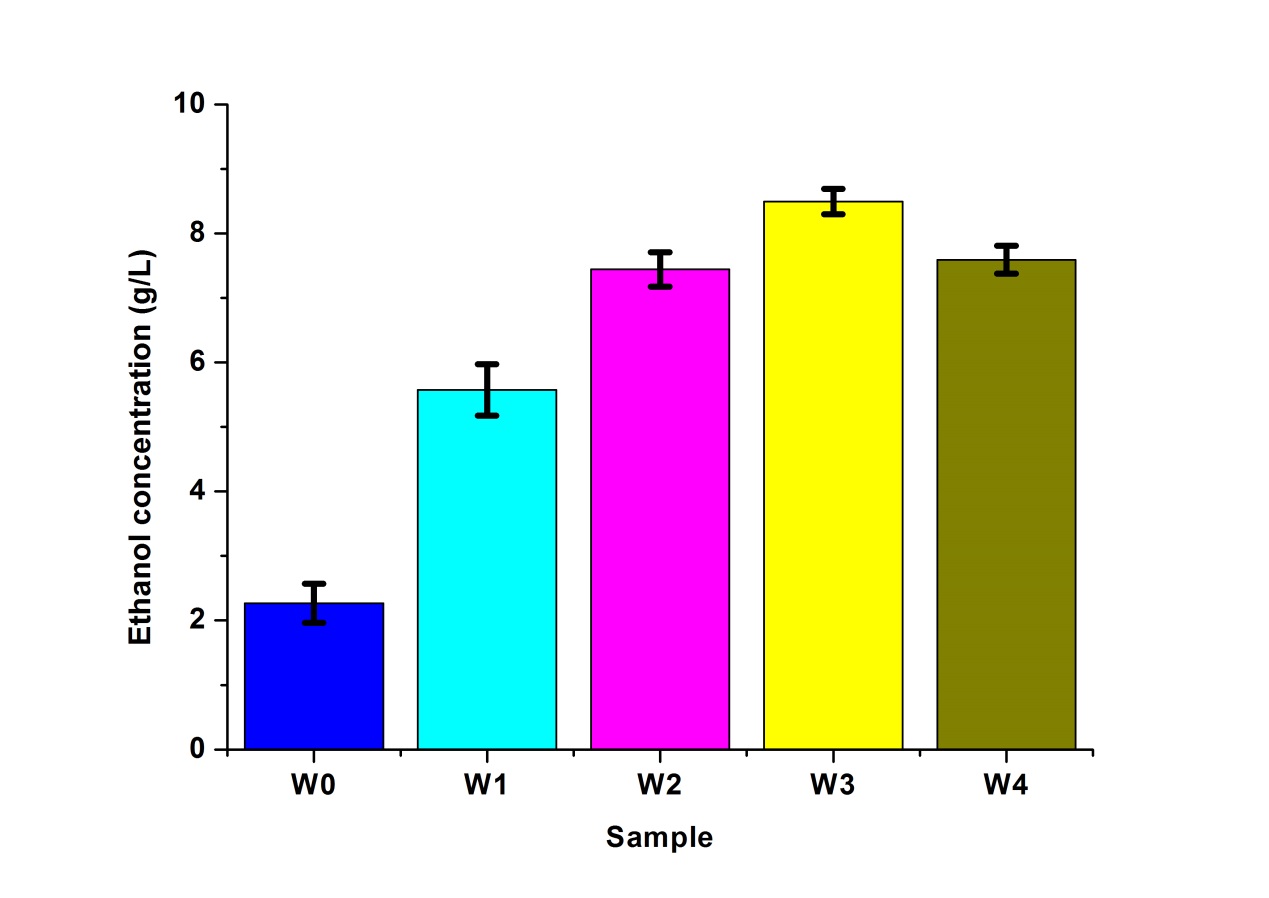


**_a)_**


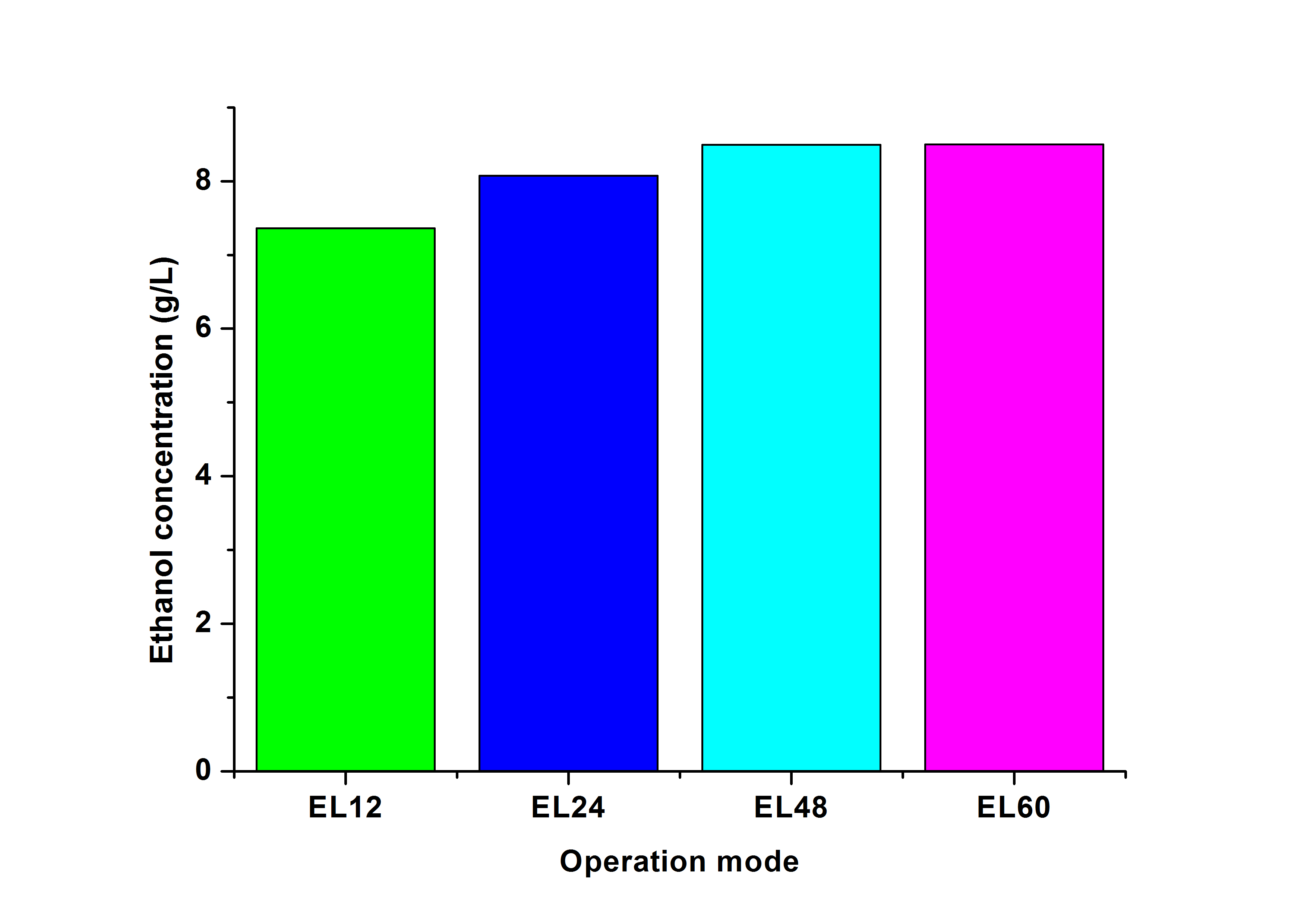


**_b)_**
